# Blood Grouping Applied to Skin Grafting

**Published:** 1918-10

**Authors:** 


### *The Principle of Blood Grouping Applied to Skin Grafting.*

H. K. Shawan, Captain, M. R. C., U. S. A. Preliminary note on a paper which will be published elsewhere.

Grafts may be classified as : (1) Autografts, where the skin is transplanted from the same individual; (2) isografts, where the skin is obtained from another person; and (3) zoo-grafts, where the graft is taken from one of the lower animals. All observers agree that the autograft is the most satisfactory. A permanent " take " is least likely and is sometimes impossible to obtain with zoo-grafts. In the case of isografts, such uncertain results have been obtained that many surgeons have been led to abandon their use.

Whether partial or total thicknesses of skin were used, a peculiar phenomenon has frequently been observed in the use of isografts. In the course of the second to the fourth week, a certain percentage of these apparently healthy primary takes begin a slight desquamation of the epidermis, then they show signs of resorption by becoming smaller, paler and thinner, and shortly are either reduced to a mere bluish pellicle or have completely disappeared. The grafts had apparently faded or melted away little by little. Having had similar experiences, and having seen the uniformly successful results obtained in blood transfusion with matched blood, it occurred to me that the failure in permanent success of isodermic grafts might be in part dependent on the same underlying principles.

Summary:

1. Autografts grow best.
2. Isografts, obtained from donors of the same blood group as the recipient, or from Group IV donors, became permanent "takes" and grew almost, if not equally, as autografts.
3. Isografts, where the donor and recipient were of different groups, did not remain as permanent growths except when Group IV skin was used or when the recipient was a member of Group I.
4. Group I grew permanent skin from donors of all of the four groups and apparently equally well.
5. Group IV skin grew permanently on recipients of all groups, but only isografts and autografts remained as permanent "takes" on them.
6. It appears that skin grafting obeys the principle of blood grouping, as in the transfusion of blood.

